# Pressing Need at the Front for Mufflers

**Published:** 1917-01-20

**Authors:** John Penoyre

**Affiliations:** 8 King's Bench Walk, Inner Temple, E.C.


*To the Editor of* THE HOSPITAL.

### Pressing Need at the Front for Mufflers.

*To the Editor of* THE HOSPITAL.

SIR,—It has been my good fortune to transmit to the men in the field some 32,000 sweaters since the war broke out. Sir Edward Ward, the Director-General of Voluntary Associations, now asks if, without letting the sweater industry go down, I could "do the same for the men's mufflers, of which a very great quantity are wanted at once."

The sweater pattern, easy and economical, is to be had here for asking, but the War Office formula for mufflers is so short that I hope you may find room for it at once. The muffler should measure 58 in. by 10 in., and be made on two No. 7 needles, taking 10 oz. of fairly thick drab or khaki wool.

One knows of the enormous amount of well-considered work that has been done for the men all over the country. I feel, however, that one has but to name the incredible numbers that our armies have recently reached to justify asking this further effort. I am authorised, then, to state that the need for sweaters, mufflers, and all other hallmarked comforts is great and immediate, and that these should be sent either to the Voluntary Organisations Depôts throughout the country or to the D.G.V.O.'s Depôt at 45 Horseferry Road, S.W., or to me as below.—Yours faithfully,

JOHN PENOYRE.

8 King's Bench Walk, Inner Temple, E.C.,
January 8, 1917.

